# Molecular dynamics simulations: from structure function relationships to drug discovery

**DOI:** 10.1186/s40203-014-0004-8

**Published:** 2014-11-21

**Authors:** Pramod C Nair, John O Miners

**Affiliations:** Department of Clinical Pharmacology, Flinders University School of Medicine, GPO Box 2100, Adelaide, SA 5001 Australia

**Keywords:** Molecular dynamics simulations, Cytochrome P450, Drug-drug interactions, Genetic polymorphism, Drug design, Allosteric binding sites, Cryptic binding sites

## Abstract

Molecular dynamics (MD) simulation is an emerging *in silico* technique with potential applications in diverse areas of pharmacology. Over the past three decades MD has evolved as an area of importance for understanding the atomic basis of complex phenomena such as molecular recognition, protein folding, and the transport of ions and small molecules across membranes. The application of MD simulations in isolation and in conjunction with experimental approaches have provided an increased understanding of protein structure-function relationships and demonstrated promise in drug discovery.

## Purpose

This perspective highlights the importance of MD simulations in the area of structure function relationships (using the cytochromes P450 as an example) and drug discovery.

## Main text

### Perspective

Molecular Dynamics (MD) simulations are computational approaches based on Newton’s equations of motion and statistical mechanics principles that relate the motions and distributions of atoms and molecules. The equations of motions are solved numerically for a system of interacting atoms to extract numerous chemical and biophysical properties from the atomic data (van Gunsteren and Berendsen 1990). The key feature of this *in silico* technique is the possibility to mimic *in vitro* and *in vivo* conditions. For example, the protein may be simulated at varying pH, in the presence of water molecules and ions, with different salt or ionic concentrations, and even in the presence of lipid bilayer and other cellular components.

In MD simulations the forces between atoms and the potential energy of the system are defined by molecular mechanics biomolecular force fields. These biomolecular force fields are parameterized to fit quantum-mechanical calculations and experimental spectroscopic data. Parameterization involves definition of chemical bonding, atomic angles and dihedral angles, along with determination of partial atomic charges for calculation of the electrostatic-interaction energies, identification of appropriate van der Waals atomic radii, etc. A number of biomolecular force fields are available for simulations, the most common being AMBER (Cornell et al. 1995), CHARMM (MacKerell et al. 1998), and GROMOS (Oostenbrink et al. 2004). The potential energy function used in the GROMOS96 (Oostenbrink et al. 2004) force field is given in equation (1). Although these biomolecular force fields differ in the way they are parameterized, the results obtained from each are generally similar. Some of the commonly used softwares for MD simulations include GROMACS (Van Der Spoel et al. 2005), NAMD (Phillips et al. 2005), AMBER (Case et al. 2005), and CHARMM (Brooks et al. 2009).

1 $$ \begin{array}{l}V\left({r}_1,{r}_2,\cdot \cdot \cdot, {r}_N\right)={\displaystyle \sum_{bonds}\frac{1}{4}}{K}_b{\left({b}^2-{b_0}^2\right)}^2+\\ {}\kern3.96em {\displaystyle \sum_{angles}\frac{1}{2}}{K}_{\theta }{\left( \cos \uptheta - \cos {\theta}_0\right)}^2+\\ {}\kern3.72em {\displaystyle \sum_{impropers}\frac{1}{2}{K}_{\xi }{\left(\xi -{\xi}_0\right)}^2+}\\ {}\kern3.72em {\displaystyle \sum_{dihedrals}{K}_{\phi }{\left[1+ \cos \left(\delta \right) \cos \left(m\phi \right)\right]}^2}+\kern5.4em \\ {}\kern4.08em {\displaystyle \sum_{pairs}\left(\frac{C{12}_{ij}}{{r_{ij}}^{12}}-\frac{C{6}_{ij}}{{r_{ij}}^6}\right)}+\\ {}\kern3.84em {\displaystyle \sum_{pairs}\frac{q_i{q}_j}{4\pi {\varepsilon}_0{\varepsilon}_1}}\frac{1}{r_{ij}}\\ {}\kern2.16em \end{array} $$

Eq. 1 In this function, *V* is the potential energy, *r* is the position of the particle (coordinate) at a particular time, *b* is the bond length, *b*_*0*_ is the bond length at equilibrium, and *k*_*b*_ is the bond force constant; *θ* is the bond angle, *θ*_*0*_ is the bond angle at equilibrium, and *k*_*θ*_ is the bond angle force constant; *ξ* is the improper dihedral angle, *ξ*_*0*_ is the improper dihedral angle at equilibrium, *k*_*ξ*_ is the force constant for improper dihedral; *φ* is the dihedral, *φ*_*0*_ is the dihedral angle at equilibrium, *k*_*φ*_ is the force constant for torsional dihedral interactions; *r*_*ij*_ is the distance between particles *i* and *j*, C12 and C6 are Lennard-Jones parameters; *ε*_0_ is the dielectric permittivity of vacuum, *ε*_1_ is the relative permittivity of the medium in which the particles are embedded, and *q*_*i*_ and *q*_*j*_ are the charges of particles *i* and *j*, respectively.

The spatiotemporal resolution attained at a particular timescale and the biomolecular processes that can be captured are important considerations in MD simulations. Simulating a system over the time scale 10^−12^ s - 10^−9^ s (picoseconds to nanoseconds) can identify motions such as atomic fluctuations, conformational changes in amino acid side chains and loop motions. However, to understand more complex biomolecular events such as large domain motions, protein folding, protein-ligand binding and the transport of molecules across membranes, simulation timescales of microseconds to milliseconds are required (Dror et al. 2012). The first molecular dynamics simulation of a biomacromolecule of therapeutic interest was published almost four decades ago. This simulation was performed in a vacuum with simple molecular mechanics parameters for a short time (9.2 ps) (McCammon et al. 1977). Since then, several advancements have taken place both in terms of computational power and algorithms. For instance, a recent two-millisecond time scale MD simulation of the β_2_-adrenergic receptor using Google’s Exacycle cloud-computing platform has led to a detailed understanding of multiple activation pathways and differential interactions of agonists and inverse agonists (Kohlhoff et al. 2014). This demonstrates the application of MD simulations for investigating complex biological phenomenon.

The importance of simulation techniques arises from the fact that biomacromolecules such as proteins exist in a dynamic state of motion. These dynamic motions are essential for the specific functions of biomacromolecules such as intermolecular protein-binding interactions or downstream signalling. Moreover, dynamic motions among the molecules are key driving forces for biomolecular events including molecular self-assembly, dimerization/oligomerization, and adaptive conformational changes on ligand binding or the transport of drugs and ions across channels and cellular membranes. Even though multiple experimental techniques aid the understanding of the structural features of biomolecules, they fail to characterize dynamic motions. MD simulations provide a means to model the flexibility and conformational changes in molecules at an atomic level and thus explore areas that are difficult to characterize experimentally.

### Structure Function Relationships

X-ray crystallography is the most widely used experimental technique employed for elucidating the 3-dimensional (3D) structures of biomolecules. The underlying principle of this technique is that a periodic crystal with an ordered arrangement of the atoms can diffract x-ray beams. A diffraction pattern, which is characteristic to the particular arrangement of atoms in a crystal, is then generated using a series of mathematical calculations. Further calculations leads to the generation of electron densities, which are then used to build the 3D structure of the molecule based on atomic coordinates. However, structures derived by x-ray crystallography can only provide a snapshot of one particular conformational state of a biomolecule. For instance, in the case of human cytochromes P450 (CYP), which comprise a superfamily of enzymes responsible for the metabolism of drugs, non-drug xenobiotics and endogenous compounds (Miners et al. 1988, Miners and Birkett 1998), x-ray crystallographic techniques have provided invaluable insights into the secondary and tertiary structures of CYP enzymes (Yano et al. 2004). However, it is now evident from the approximately 150 human cytochrome P450 crystal structures that have been elucidated to date that CYP proteins are highly flexible and show dramatic structural adaptability in the presence of a ligand. For example, the experimental structure of CYP3A4 shows an active site volume of 950 Å^3^ in the ligand-free state, 1650 Å^3^ when bound to ketoconazole and 2,000 Å^3^ when bound to erythromycin (Ekroos and Sjögren 2006). To understand such dramatic changes in the active site architecture associated with the dynamic behavior of P450s, MD simulations provide a more practical alternative than the generation of x-ray crystal structures for every enzyme-ligand combination. In recent years, several applications have shown the potential of MD simulations for providing insights into structural aspects of human cytochromes P450, including demonstration of the plasticity of the substrate binding site (eg. Hendrychova et al. 2012) and identification of solvent and ligand (access and egress) channels (eg. Schleinkofer et al. 2005). Recent MD simulation studies have also demonstrated ligand cooperativity and allosteric binding sites associated with certain CYP enzymes (Li et al. 2011, Bren and Oostenbrink 2012). This demonstrates the potential of MD simulation for understanding drug-drug interactions arising from CYP enzyme inhibition or activation at an atomic level. Moreover, MD simulations can be used to investigate altered conformational states of CYP proteins that result from genetic polymorphism.

### Drug Discovery

In the recent years, the ability to design drugs rationally based on the structures of the protein targets has been a successful strategy in drug discovery. The so-called structure based drug design (SBDD) approach involves the design of molecules that can specifically bind to the key structural domains (binding pocket(s)) of the protein target (e.g. Varney et al. 1992, Dorsey et al. 1994). The aim is to modulate the activity of the protein in order to alleviate disease progression. For instance, designing molecules that can maximize the complementary interaction at the active site of the target receptor can be a useful strategy in the structure guided inhibitor design (Varney et al. 1992, Dorsey et al. 1994). However, recent studies have shown the importance of MD simulation to investigate the biomolecular flexibility associated with ligand recognition (Nair et al. 2011, Nair et al. 2012). Studying the flexibility of the target receptor would thus permit the improved design of drugs over the simplistic lock and key conceptualization of the static receptor. Also, simulations allow the exploration of additional druggable sites (cryptic or allosteric) on the target receptor that is not evident from experimental (e.g. X-ray) structures (Frembgen-Kesner and Elcock 2006). An example is the identification of a novel binding trench in HIV-integrase through dynamic simulation (Schames et al. 2004). This led to the discovery of raltegravir, the first of a new class of anti-HIV (integrase inhibitor) drugs (Summa et al. 2008). Similarly, a novel transiently open binding pocket was identified recently using MD simulation of the p53 protein, which has potential application for the design of novel anticancer drugs (Wassman et al. 2013). Recent MD simulations have also shown the likelihood of identifying allosteric binding sites in the human β_1_ and β_2_ adrenergic receptors (Ivetac and McCammon 2010).

## Conclusion

There has been a dramatic increase in our understanding of disease states and therapeutic targets over the last two decades. With the current bioinformatics applications and sequencing data it is likely that the number of putative drug targets will continue to increase in the coming years, as in the case of G-protein coupled receptors. With increased computing power and continued developments in the efficiency of simulation codes and faster algorithms, the future of *in silico* approaches is promising. Molecular dynamics simulations are likely to play an increasingly important role for understanding the structure function relationships of pharmacological targets and in the development of novel therapeutics.
